# Prof Martin Fortin, An appreciation

**DOI:** 10.1177/26335565251323250

**Published:** 2025-02-25

**Authors:** Susan M Smith, Frances S Mair, Marjan van den Akker

**Affiliations:** 1 8809 Trinity College Dublin, Dublin, Ireland; 2 Institute of Health and Wellbeing, 3526 University of Glasgow, Glasgow, UK; 3 9173 Institute for General Practice, Goethe University Frankfurt, Frankfut am Main, Germany

Prof Martin Fortin was one of the founding Editors-In-Chief of the Journal of Comorbidity which subsequently evolved into the Journal of Multimorbidity and Comorbidity. He has retired and stepped down from his position with the Journal and we want to acknowledge the enormous impact he has had in the field of multimorbidity research.

Prof Fortin began his career in 1985 in a small, isolated village, working with a small medical team for two years. He then transitioned to practicing in a mid-sized town, still far from major urban centers, where he took on various responsibilities. After about 12 years in clinical practice, he completed a Master’s degree in Epidemiology and embarked on a research career around the year 2000.

In 2007, he was awarded a Research Chair on Chronic Diseases in Primary Care at the Université de Sherbrooke (Quebec), Canada, a position he held until 2019. His primary research focus has been on the epidemiology of multimorbidity and the development of innovative interventions to better equip frontline professionals in managing patients with multimorbidity. He was one of the first clinician researchers to address “Multimorbidity’s many challenges” in an editorial in the British Medical Journal in 2007, co-written with our other founding Co-Editor in Chief, Prof Marjan Van Den Akker. Prof Fortin’s research has led to over a hundred peer-reviewed publications on multimorbidity, which are internationally cited.

Alongside his research career, he held several administrative positions, including Clinic Director, Director of University Research within a healthcare organization, and President of the Researchers' Section of the College of Family Physicians of Canada.

He assumed the leadership of the International Research Community on Multimorbidity since its creation in 2007 until the leadership was transferred to colleagues in the University of Glasgow in 2021.

Prof Fortin has also been recognized by his peers through numerous awards for excellence in research. In 2017, he was inducted as a member of the **Canadian Academy of Health Sciences.** More recently, he was awarded **Researcher of the Year** by the **College of Family Physicians of Canada in 2022**.

Prof Fortin retired in July 2022, from the Université de Sherbrooke where he was professor since 1998. His clinical work then shifted from general practice to public health and he retired completely June 1^st^ 2024.

On a personal basis, Prof Fortin is a father of five and grandfather of four. He is a runner and his favorite distance is half-marathon which he has run several times in the past five years. He has also run two marathons , the last one being in October 2024, in the Laurentians, North of Montreal. His new passion is birding and photography and he intends to do more of this during his retirement.

We have been colleagues of Prof Fortin for many years and we will greatly miss his wisdom, expertise and enthusiasm in the Journal of Multimorbidity and Comorbidity. He has left an enduring legacy, and the Journal is committed to building upon it by publishing high-quality research that strengthens the evidence base to support healthcare delivery and improve health outcomes for individuals living with multimorbidity.

